# Resolving large‐scale pressures on species and ecosystems: propensity modelling identifies agricultural effects on streams

**DOI:** 10.1111/1365-2664.12586

**Published:** 2016-01-18

**Authors:** Caitlin E. Pearson, Steve J. Ormerod, William O.C. Symondson, Ian P. Vaughan

**Affiliations:** ^1^ Catchment Research Group Cardiff School of Biosciences Cardiff University Cardiff CF10 3AX UK

**Keywords:** farming, land cover, land‐use policy, macroinvertebrates, monitoring data, physicochemical effects, propensity modelling, rivers

## Abstract

1. Although agriculture is amongst the world's most widespread land uses, studies of its effects on stream ecosystems are often limited in spatial extent. National monitoring data could extend spatial coverage and increase statistical power, but present analytical challenges where covarying environmental variables confound relationships of interest.
2. Propensity modelling is used widely outside ecology to control for confounding variables in observational data. Here, monitoring data from over 3000 English and Welsh river reaches are used to assess the effects of intensive agricultural land cover (arable and pastoral) on stream habitat, water chemistry and invertebrates, using propensity scores to control for potential confounding factors (e.g. climate, geology). Propensity scoring effectively reduced the collinearity between land cover and potential confounding variables, reducing the potential for covariate bias in estimated treatment–response relationships compared to conventional multiple regression.
3. Macroinvertebrate richness was significantly greater at sites with a higher proportion of improved pasture in their catchment or riparian zone, with these effects probably mediated by increased algal production from mild nutrient enrichment. In contrast, macroinvertebrate richness did not change with arable land cover, although sensitive species representation was lower under higher proportions of arable land cover, probably due to greatly elevated nutrient concentrations.
4. *Synthesis and applications*. Propensity modelling has great potential to address questions about pressures on ecosystems and organisms at the large spatial extents relevant to land‐use policy, where experimental approaches are not feasible and broad environmental changes often covary. Applied to the effects of agricultural land cover on stream systems, this approach identified reduced nutrient loading from arable farms as a priority for land management. On this specific issue, our data and analysis support the use of riparian or catchment‐scale measures to reduce nutrient delivery to sensitive water bodies.

## Abstract 2

Propensity modelling has great potential to address questions about pressures on ecosystems and organisms at the large spatial extents relevant to land‐use policy, where experimental approaches are not feasible and broad environmental changes often covary. Applied to the effects of agricultural land cover on stream systems, this approach identified reduced nutrient loading from arable farms as a priority for land management. On this specific issue, our data and analysis support the use of riparian or catchment‐scale measures to reduce nutrient delivery to sensitive water bodies.

## Introduction

With global agricultural production set to double between 2005 and 2050 (Tilman *et al*. 2011), the reconciliation of food production and environmental protection is a key challenge for sustainable development (Robertson & Swinton 2005). The difficulties of balancing the use and protection of natural resources were evident in the expansion of UK agriculture between 1940 and 1980, as intensification resulted in habitat simplification, environmental pollution and declines in a broad range of terrestrial and freshwater taxa (Robinson & Sutherland 2002). Seen from an ecosystem perspective, agricultural services were gained at the potential expense of other ecosystem services including carbon sequestration, water quality regulation and nutrient cycling (Dale & Polasky 2007).

The effects of agriculture on freshwaters are of particular interest due to the conservation, economic and cultural importance of these systems (Dudgeon *et al*. 2006). The ecosystem services provided by streams, including water supply, fisheries and recreation, can be impacted on by the effects of both arable and intensive pastoral land uses, the latter where high densities of livestock graze on fertilized and reseeded pasture (hereafter ‘improved pasture’). The mechanisms involved include altered flow regimes (Niyogi *et al*. 2007), increased nutrient and inorganic sediment inputs (McDowell *et al*. 2003), and altered bankside vegetation structure (Townsend *et al*. 1997). However, the effects of these combined changes on stream community structure are highly variable. For example, improved pasture land cover has been associated with both lower (Quinn & Hickey 1990; Liess *et al*. 2012) and higher invertebrate richness and sensitive species representation compared to reference grasslands (Thompson & Townsend 2004), with other studies finding no significant associations (e.g. Riley *et al*. 2003). One possible explanation for these variable results is that studies have often been of limited spatial extent and may not generalize to different regions (Knapp *et al*. 2004). This lack of generality is a common concern in ecology, where studies are often too site‐specific to guide environmental and land‐use policies at the national or regional scales over which they are implemented (Donald *et al*. 2006).

Whereas national‐scale studies to assess the impacts of agricultural practices are well‐established for vertebrates such as birds (e.g. Chamberlain *et al*. 2000; Donald *et al*. 2006), they are lacking for most other taxa, probably reflecting the difficulties of obtaining large‐scale data. Fortunately, many nations have extensive environmental monitoring programmes and high‐resolution land‐cover imagery that could redress this gap. In England and Wales, for example, river monitoring data comprise records of water chemistry, macroinvertebrates and geomorphology from thousands of locations. These data provide an opportunity for large‐scale analyses within realistic budgets and time frames, whilst the statistical power afforded by the large sample sizes makes them a valuable adjunct to traditional field surveys (Vaughan & Ormerod 2010). Beyond basic statutory reporting, however, there have been surprisingly few attempts to utilize these data to address large‐scale ecological questions (e.g. Murphy & Davy‐Bowker 2005; Vaughan & Ormerod 2012).

A second challenge for research across large spatial extents is that there is often a complex pattern of collinearity between the variable of interest and other environmental variables. Across England and Wales, agricultural land cover correlates with environmental characteristics such as geology, soil type and climate, and trying to distinguish the impacts of agriculture is a major challenge (Schriever *et al*. 2007). Multiple regression is commonly used to investigate the effects of land use and attempts to control for these covariates. However, collinearity between the covariates and the variable of interest, or amongst covariates, can bias the estimated effect sizes and lead to unstable coefficient estimates with large standard errors, whilst complex relationships between the covariates increase the risk of model misspecification (Graham 2003).

Fields including medicine, economics and social sciences face similar challenges in trying to quantify effect sizes and determine causal relationships from survey data, leading to the development of propensity score approaches (Dehejia & Wahba 2002). The propensity approach attempts to mimic randomized controlled experiments by comparing the effect of the ‘treatment’ (e.g. different land cover) in subsamples of the full data set that are closely matched on background covariates (e.g. climate, geology). This comparison is commonly achieved by building a regression model to predict the probability or size of the ‘treatment’ based on the background covariates and subdividing the data set into a small number of groups which have similar predictions (termed propensity scores): hence a similar distribution of the environmental covariates (Rosenbaum & Rubin 1983). Within each group, the correlations between the covariates and the treatment are much weaker and so the effect of the treatment on response variables of interest can be modelled with reduced potential for confounding (Rosenbaum & Rubin 1983). Both simulation and empirical studies have shown that the propensity approach can minimize bias in regression coefficients and allow changes in response variables to be ascribed more directly to the causal effect of the treatment variable (e.g. Dehejia & Wahba 2002; Imai & Van Dyk 2004). Propensity scoring could be of great value to ecology, yet has been largely ignored with the notable exceptions of Yuan (2010), Bottrill *et al*. (2011) and Chessman (2013).

Here, we used the propensity approach to analyse the effects of agricultural land cover on in‐stream habitat, water chemistry and invertebrate community structure across England and Wales, making this one of the most comprehensive assessments of broadscale agricultural effects, and the first application of propensity modelling to assess the effects of land cover – a subject well known for the challenges of collinearity (Van Sickle 2003). In the highly modified UK landscape, there is little scope to compare agricultural land uses with semi‐natural land cover or catchments that differ only in terms of a focal land‐cover type. Instead, we compared streams with differing proportions of pastoral or arable land cover within their catchments or riparian zones against a background mix of other land covers that typically occur within the same propensity score group. This comparison will indicate what the effects of contemporary changes in catchment land cover could be, that is, the effect of increasing arable or pastoral cover relative to other land uses within the catchment. We aimed to quantify the effects of varying agricultural land cover at the national scale with characteristics that describe the physicochemical conditions and biological structure of stream ecosystems. Changes in these characteristics would indicate alteration to ecosystem functioning with potential consequences for ecosystem service provision. Specifically we tested the hypotheses that increasing improved pastoral or arable land cover at the national scale would:

1. Increase nitrate and phosphate concentrations, stimulating increased in‐stream vegetation.
2. Increase sediment deposition.
3. Simplify bankside vegetation.
4. Lower invertebrate family richness and representation of taxa sensitive to organic pollution or low dissolved oxygen.
5. Decrease the diversity of macroinvertebrate functional feeding guilds indicating the potential for impaired ecosystem functioning (Larsen & Ormerod 2010).

## Materials and methods

### Physical Habitat Data

River Habitat Survey (RHS) is the standard method for recording the physical characteristics of rivers and streams in England and Wales (Seager *et al*. 2012), covering channel morphology, bed and bank materials, flow types, vegetation in the channel and on the banks, surrounding land use and anthropogenic modifications at ten equidistant ‘spot checks’ along a 500‐m reach. The extent of features over the reach and presence of any additional features is recorded in a ‘sweep‐up’ assessment (see Environment Agency 2003 for a detailed description of the method). A national baseline survey was conducted in England and Wales during 2007–2008, with three reaches randomly selected within each 10‐km Ordnance Survey grid square in England and Wales (Seager *et al*. 2012; Fig. S1, in Supporting Information).

Five response variables were derived from RHS data to capture key river characteristics that were hypothesized to be affected by agriculture (Tables 1 and S2). Due to severe skews and U‐shaped distributions, the response variables were dichotomized (Tables 1 and S2; Vaughan, Merrix‐Jones & Constantine 2013). Rerunning analyses with alternative category thresholds confirmed that results were not sensitive to the precise thresholds selected (Table 1).

**Table 1:** Explanation of response variables derived from River Habitat Survey data. Each site was categorized as Yes or No for each of the response categories

Habitat characteristic	Response variable	Alternative category thresholds
Riparian Bankside trees	≥50% of spot checks with broadleaf woodland within 5 m of bank top	≥40% and ≥60% of spot checks
Macrophytes	≥1 spot check with submerged, amphibious, emergent, rooted or floating‐leaved vegetation or reeds	≥2 spot checks
Filamentous algae	≥1 spot check with filamentous algae	≥2 spot checks
Silt/sand deposits	≥1 spot check with sand and silt substrate	≥2 spot checks
Sediment storage	Presence of point, side or mid‐channel bars	

### Macroinvertebrate and Water Chemistry Data

Macroinvertebrate and water chemistry data were collected during routine monitoring by the Environment Agency in 2006. This year had a large sample size and was temporally consistent with the RHS data (2007–2008) and land‐cover imagery (2007; described below). Sampling sites were identified where water chemistry and biology were recorded within 500  m of each other and monthly chemistry samples taken over the year preceding the invertebrate sample. To minimize the risk of spatial autocorrelation only one site per tributary was retained for analysis (*n* = 955, Fig. S1). Macroinvertebrates were collected using standard 3‐min kick samples and identified to family (Murray‐Bligh 1999). Presence/absence data from spring (March–May) and autumn (September–November) 2006 macroinvertebrate samples were combined and family richness and average score per taxon (ASPT) calculated for each site (Table S3, Supporting Information). ASPT is a standard measure of community sensitivity to organic pollution calculated by ascribing each family a score between 1 (tolerant) and 10 (highly sensitive) based on expert opinions and averaging this score across all families present in a site (Armitage *et al*. 1983).

Each family was assigned an affinity for different functional feeding guilds (FFGs) based on its morpho‐behavioural methods of food acquisition, converting the species‐level data of Schmidt‐Kloiber & Hering (2012) to family‐level using the method of Vaughan & Ormerod (2014). For each site the diversity of FFG affinities was calculated using Simpson's diversity index, producing a score between 0 and 1 where low values indicate dominance by a few feeding guilds whilst high scores indicate equitability across feeding guilds (Larsen & Ormerod 2010; Table S3).

Water chemistry data were used to indicate the influence of agricultural land cover on nutrient loading. The response variables were total oxidized nitrogen (abbreviated as nitrate because where both were recorded, nitrate approximated >99% of total oxidized nitrogen) and orthophosphate, analysed using standard methods (Standing Committee of Analysts 1987, 1992; Table S3). Annual medians were calculated for the twelve months preceding the 2006 spring invertebrate sample. Where ≥50% of these values were below detection limits, medians were estimated using regression on order statistics in R's NADA library (Lee & Helsel 2005).

### Catchment Land Cover

The proportions of arable and improved pasture land cover were determined for each RHS and invertebrate/water chemistry survey site from the 25‐m resolution UK Land cover Map 2007 (Morton *et al*. 2011). Catchment boundaries for each site were estimated from a 50‐m resolution digital elevation model (Ordnance Survey Landform Panorama) using hydrotools (v.9; Centre for Research in Water Resources, University of Texas, TX, USA) in arcinfo v. 10 (ERSI, Redlands, USA). The percentage of the catchment and the percentage of an upstream riparian strip (50 m either side of the channel for whole upstream network) under each land cover were determined using the Geospatial Modelling Environment (Beyer 2005; Tables S1–S3).

### Statistical Analysis

Propensity modelling involved four basic stages (Rosenbaum & Rubin 1983): (i) creating a model to predict the proportion of each site's catchment area under arable or improved pasture land cover from locational, climatic and geological variables; (ii) stratifying the data set into groups with similar predicted proportions of arable or pasture land cover; (iii) modelling the effect of agricultural land cover on response variables of interest within each propensity group; (iv) calculating the average effect size and 95% confidence limits across all groups, weighted by the number of observations in each group.

Four propensity models were built to predict the percentage cover: one each for arable and pastoral, in the entire catchment and in the riparian strip. All site locations (RHS and invertebrate/water chemistry) were pooled for the propensity modelling (*n* = 3135). We identified a range of potential confounding variables that covary with land cover on a national scale: slope and altitude, mean annual rainfall (mm) and temperature (°C), underlying solid geology, predominant soil texture and proportional catchment cover of urban land use and other agricultural land use (i.e. arable land for improved pasture models and *vice versa*; Table S4). Climatic variables were derived from the 1961–1990 climatic averages mapped at 5‐km resolution (UK Meteorological Office; Perry & Hollis 2005). Geological and soil data were simplified from 1:625k geological maps (British Geological Survey, 2007) into five lithological classes: hard (igneous and metamorphic), chalk, limestone, sandstone and other sedimentary (Emery *et al*. 2003) and four soil classes: loam, clay, sand and ‘other’, to reduce overfitting of the model. For all variables the mean value or the predominant category within the catchment/riparian strip was used as the predictor value. Generalized additive models (GAMs), using R's mgcv library, were used to describe the relationship between treatment land‐cover proportions and the predictor variables. Easting and Northing were also included using a tensor product smooth (Wood 2006). As the relative influence of different covariates was not of interest, the models were not checked for collinearity, nor was model simplification implemented (Harrell 2001). Predictions were made for all sampling sites using each of the four models, to give the respective propensity scores (Table S1).

For each treatment land cover (arable/pasture, catchment/riparian strip), the data were split into five equally sized groups (‘propensity groups’) using the quintiles of the predicted probabilities (Rosenbaum & Rubin 1983) and then separated into RHS and biology/chemistry data sets (Table S5). Although Rosenbaum (2002) states that five groups based on quintiles are appropriate for most data sets, all analyses were conducted with four and six groups to check that the number of propensity groups did not alter the conclusions (Tables S6 and S7).

Generalized linear models (GLMs) (binomial error distributions for RHS data) were used to describe the relationship between each response variable and percentage treatment land cover within each propensity group. Water chemistry variables were log transformed to meet model assumptions. The covariates used in the propensity model were also included in each model to account for remaining within‐group variability and to allow any covariates that strongly influence the response variable to contribute to the model (Robins & Rotnitzky 2001). Plots of residuals were used to check the model fits, alongside semivariograms (gstat library; Pebesma 2004) to ensure that there was no residual spatial autocorrelation. For each response variable, the mean effect size across propensity groups was calculated, weighted by the proportion of observations within each subclass (Imai & Van Dyk 2004). The effect sizes represent the change in the response variable for 1% increases in percentage agricultural land cover. For binomial models of habitat features these effect sizes are the odds ratios: values <1 show a decrease in likelihood and >1 an increase. A 95% confidence interval was calculated, over all *k* groups, according to eqn 1 (Benjamin 2003; Guo & Fraser 2014):

(eqn 1) $$\text{CI}=1.96\sqrt{\left(\sum_{k=1}^{k}{{\text{SE}}_{k}}^{2}{\left(\frac{n_{k}}{N}\right)}^{2}\right)}$$

where SE = standard error of group estimate*, n * = number of observations in group, *N* = total number of observations. Given the number of response models (20 for each of invertebrate/chemistry data and RHS data) confidence limits were extended using the method of Benjamini & Yekutieli (2005) to control for the false discovery rate. Effects were considered statistically significant (at α = 0·05) if the interval did not span zero (invertebrates and water chemistry variables) or one for the odds ratios (RHS variables).

### Evaluating the Propensity Approach

In the final stage, the propensity scoring approach was compared to conventional multiple regression (hereafter the ‘direct approach’). GLMs were fitted between percentage treatment land cover and each of the response variables, using the same covariates as for the propensity scores. The efficacy of the propensity approach was evaluated by assessing the degree to which it had reduced confounding between the treatment land cover and covariates in response models compared to the direct regression approach. To achieve this, commonality analysis was performed for each response model in the ‘yhat’ package in R (Nimon, Oswald & Roberts 2013) to give the unique and common contribution of each independent variable to the variance explained by each model. Commonality coefficients were averaged across the five propensity group models for each response variable to indicate the degree of confounding and compared to those from equivalent direct models using a paired *t*‐test.

## Results

### Propensity Models

The proportion of agricultural land cover in the riparian strip and whole catchment were strongly correlated (Pearson's *r* = 0·78 for improved pasture and *r *=* *0·86 for arable). The arable land‐cover models explained 76% of the variation at the catchment scale and 64% within the riparian strip, and the mean correlation between land use and the environmental covariates was 58% lower within propensity groups compared to the entire data set in both cases (Table S5). At both scales, the predicted proportion of arable land cover increased as the proportion of improved pasture and urban land use decreased, as altitude and rainfall decreased and towards the east on chalk geology with loamy soils (Fig. S2). Improved pasture was less predictable: models explained 45% of the variation at the catchment scale and 36% within the riparian strip. For the majority of covariates the correlation with improved pasture across the whole data set was low, but was still reduced by 24% (catchment) and 55% (riparian strip) by the propensity approach (Table S5). The predicted proportion of improved pasture land cover in the catchment and riparian strip was higher in the south‐west, and increased as the proportion of arable and urban land cover decreased, and as temperature, altitude and rainfall decreased (Fig. S3).

### Effects of Agriculture based on the Propensity Approach

**Figure 1:**
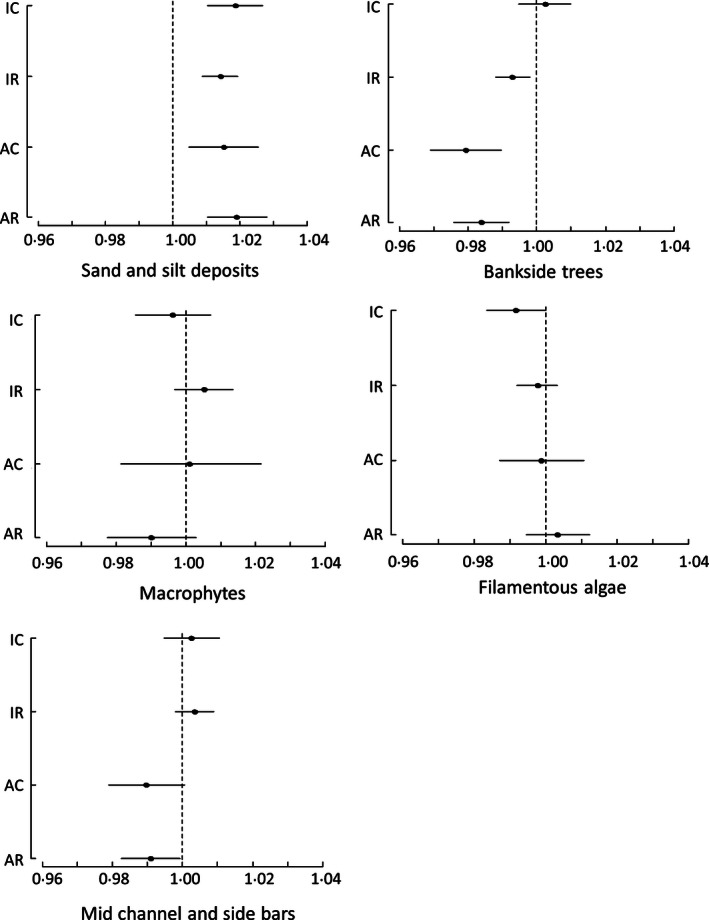
Changes in the likelihood of occurrence (odds ratios) of habitat characteristics, based on the propensity approach, for each percentage increase in the proportion of the treatment land covers: improved pasture in the catchment (IC), improved pasture in riparian strip (IR), arable farming in catchment (AC) and arable farming in riparian strip (AR). Horizontal bars show 95% confidence intervals across the five propensity groups. Values of 1 = no change.

Estimated effects of agriculture on physical habitat were similar in direction and magnitude for land cover measured at the catchment and riparian scales (Fig. 1). Sites with a higher proportion of their catchment or riparian strip under either improved pasture or arable land cover had a significantly higher likelihood of containing silt or sand deposits. Sites with a higher proportion of either land cover in the riparian strip, or a higher proportion of arable cover in the catchment, had a significantly lower occurrence of bankside trees (Fig. 1). Neither improved pasture nor arable land cover had a significant relationship with the likelihood of occurrence of macrophytes, filamentous algae or stable sediment deposits (in‐channel bars; Fig. 1).

**Figure 2:**
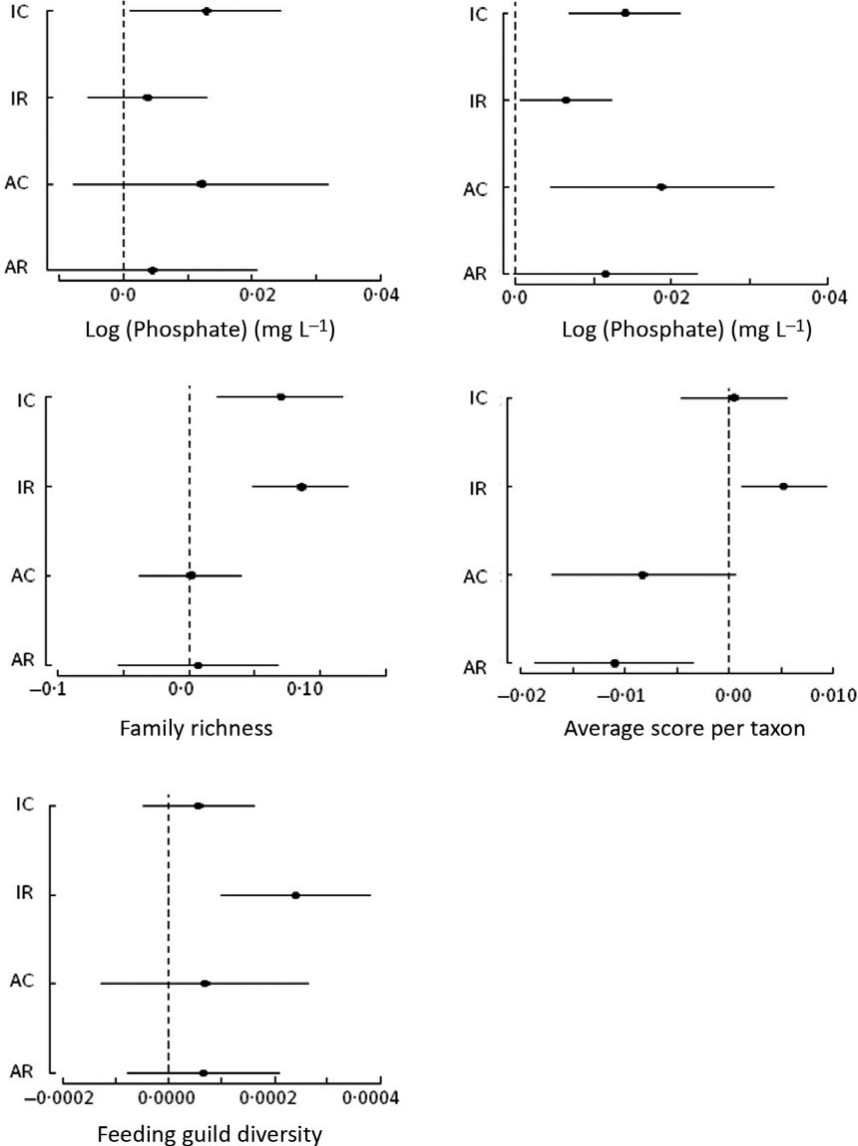
Changes in water chemistry and invertebrate community variables based on the propensity approach, for each percentage increase in the proportion of the treatment land covers, improved pasture in the catchment (IC), improved pasture in riparian strip (IR), arable farming in catchment (AC) and arable farming in riparian strip (AR). Horizontal bars show 95% confidence intervals across the five propensity group.

Phosphate concentrations showed no significant relationships with arable land cover at either spatial scale, but had a significant positive relationship with improved pasture at the catchment scale. Phosphate concentrations were 0·2 mg L^−1^ higher in catchments with 100% improved pasture cover compared to catchments with no improved pasture. Nitrate concentrations increased with both arable and improved pasture, especially when the land cover was measured at the catchment scale (Fig. 2): catchments with 100% treatment land cover were estimated to have nitrate concentrations that were 7·6 mg L^−1^ higher for improved pasture and 12·3 mg L^−1^ for arable compared to catchments with no agriculture.

**Figure 3:**
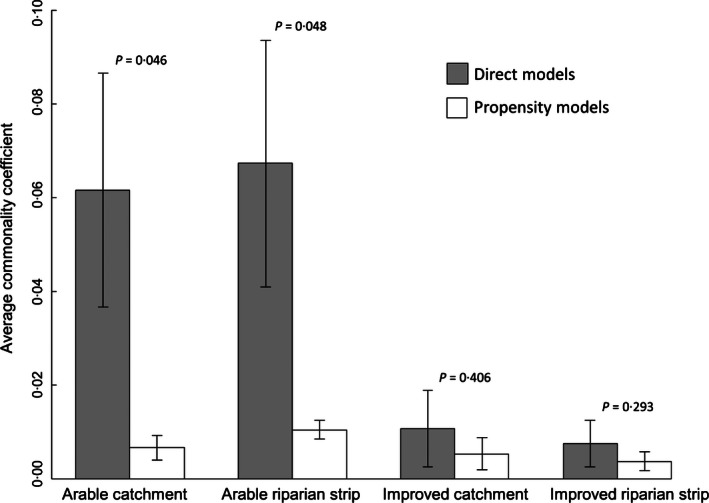
Differences in confounding between direct and propensity models. Bars show the commonality coefficients for each treatment land cover and the contribution to the regression effect that is shared with other covariates, averaged across all 10 response variables ± standard error. *P* values are the result of paired *t*‐tests comparing commonality coefficients of propensity and direct models.

Invertebrate richness increased with the proportion of improved pasture at catchment and riparian scales. The estimated effect size translated to six (catchment) or eight (riparian) extra families in sites with 100% improved pasture than in sites with no improved pasture, compared to an average of 23 nationwide in 2006 (Vaughan & Ormerod 2012). The representation of taxa sensitive to organic pollution (ASPT) increased with improved pasture cover at the riparian, but not catchment, scale (Fig. 2). Richness did not show a significant response to arable land cover at either scale, but a declining ASPT score indicated a lower representation of sensitive species, although this was only significant at the riparian scale. Although feeding guild diversity was significantly higher under improved pasture at the riparian scale the effect size was very small and there was no significant response to arable land cover (Fig. 3).

### Comparison with Direct Models

Commonality analysis showed that there was little confounding between improved pasture land cover and covariates in direct response models (Fig. 3), consistent with the low correlations between land cover and covariates across the whole data set (Table S5). Although the propensity approach did reduce the amount of variance shared between the treatment land use and covariates the magnitude of this reduction was small and insignificant (Fig. 3). The magnitude of confounding was much greater in models of responses to arable land cover. The propensity approach effectively reduced commonality coefficients across all response variables (Fig. 3). Direct models suggested that land cover had a significant effect more frequently than propensity models: 75% of the models tested compared to 45% of models using the propensity approach (Tables S2 and S3).

## Discussion

A large body of literature illustrates how land cover can affect stream ecosystems, including recent experiments that have increased mechanistic understanding of the effects of single stressors and their interactions (e.g. Matthaei, Piggott & Townsend 2010). The practical difficulties of manipulating catchment land cover experimentally, however, mean studies examining the aggregate impacts of agricultural land cover must rely on observational data. Typically, these studies compare catchments with differing land covers, matched as far as possible to other covariates. Despite minimizing differences between catchments these studies often encompass variability in confounding factors such as catchment elevation or microclimate (*e.g*. Townsend *et al*. 1997; Riley *et al*. 2003). Further, the majority of land‐use studies are restricted to small geographical areas with similar site characteristics, which may reduce their generality to other regions and limit their utility for guiding national‐scale environmental policy.

Here, national monitoring data allowed one of the largest studies of agricultural effects on stream systems to date, both in spatial extent and sample size (but see Meador & Goldstein 2003; Carlisle & Hawkins 2008). There are, however, important limitations when using monitoring data. First, there is limited detail recorded at each location; RHS data provided relatively crude measures of physical habitat (e.g. fine sediment loading), whilst invertebrate data were available only at family level, obscuring species‐level responses. The difficulties in assigning traits at the family level (cf. species or genera) may account for the lack of ecologically significant responses in feeding guild representation observed in this study. More generally, our land‐cover categories cover a range of management practices (e.g. differences in stocking density, fertilizer application and pesticide use), which may differentially affect stream ecosystems. In combination, these limitations are likely to reduce the ability to detect significant responses to land cover change and increase the uncertainty associated with the modelled effects. Despite these limitations, the unrivalled sample size and spatial coverage of these data sets makes them valuable for large‐scale assessments (Vaughan & Ormerod 2010). First, we discuss the propensity method and then the ecological implications of the findings.

### Evaluating the Propensity Approach

The benefits of propensity scoring have been confirmed by both theoretical studies and successful application in several fields, including recently in ecology (Yuan 2010; Bottrill *et al*. 2011; Chessman 2013). Propensity scores have the ability to control for a large array of covariates by combining them into a single score, whereas attempts to control covariation through experimental design are restricted to relatively few covariates (Dehejia & Wahba 2002). As we demonstrate here, grouping data by propensity scores reduces the correlations between the treatment and covariates relative to the whole data set. Therefore, compared to conventional regression models, propensity modelling (i) reduces the potential for covariate bias in estimated treatment–response relationships, (ii) increases the likelihood that treatment–response relationships can be represented by linear models, reducing the risk of model misspecification or the need for complex models and, (iii) makes models more robust to extrapolation by minimizing their reliance upon the particular distribution of the background covariates in the data set (Imai & Van Dyk 2004; Vansteelandt & Daniel 2014). Set against these advantages are the additional stage of data analysis required in propensity modelling and limited benefit when covariates are poor predictors of the treatment variable (Weitzen *et al*. 2005).

The few ecological studies to apply propensity modelling have shown an effective reduction in the strength of covariate bias (Yuan 2010; Bottrill *et al*. 2011). Here, the efficacy of the propensity approach differed between arable and improved pasture land cover. The propensity model explained much of the variation in arable land cover and effectively restricted its collinearity with other covariates within each propensity group. Thus, the variance explained by the shared effects of arable land cover and other covariates was substantially reduced; limiting bias in the coefficient estimates (Imai & Van Dyk 2004). The benefits for improved pasture were less clear, with a smaller reduction in collinearity and similar model results for propensity and direct methods. The key difference was that collinearity was much lower in the original data set, indicating less potential for confounding between pasture and environmental covariates, which may indicate that improved pasture is less closely tied to large‐scale environmental conditions in the UK than arable land cover, or that we may have overlooked important confounders. The latter seems less likely given the range of environmental covariates, alongside geographical position, that was considered. The division of ‘improved grassland’ from semi‐natural grasslands may be indistinct (Morton *et al*. 2011), whilst the distribution of reseeded grasslands may depend on decisions taken by individual land owners at smaller spatial scales than our environmental data. Whatever the reason, the propensity approach offered little advantage over traditional regression methods for improved pasture. Thus, the most obvious applications for propensity modelling will be when there is strong collinearity between the treatment variable and known environmental covariates, as frequently occurs in large‐scale ecological studies, and which are also the conditions under which controlling for such covariates is of greatest importance.

### Effects of Agricultural Land Cover on Stream Ecosystems

Whilst many studies have considered the effects of arable or pastoral land cover on streams, surprisingly few have studied both simultaneously (e.g. Kyriakeas & Watzin 2006). Our study also differed from most previous work by comparing arable and pasture to the mix of other land covers in the highly modified landscapes of England and Wales, rather than to semi‐natural ‘reference’ conditions, increasing its relevance to decisions about rural policy and changing land cover.

Invertebrate richness and sensitive species representation were higher under improved pasture, whereas sites with arable land cover had a lower representation of sensitive taxa but no change in species richness, suggesting a turnover from sensitive to tolerant families with increasing arable land cover. These results, on a national scale, are contrary to predictions and to a previous small‐scale comparison which showed lower sensitive species representation in both arable land and pasture compared to reference grasslands, with greater impacts in pasture (Kyriakeas & Watzin 2006).

As predicted, both agricultural types increased the frequency of silt/sand deposits and elevated nitrate concentrations. The change in fine sediment cover was similar for both agricultural types; a fourfold increase in the odds of occurrence between sites with 0 and 100% agricultural land cover. The impact of this increase on invertebrates will depend on the initial sediment cover but as sensitive families have been shown to decline at a sediment threshold of 20% cover (Burdon, McIntosh & Harding 2013) the estimated increase in fine sediment has the potential to have detrimental effects on invertebrate communities.

Nutrient enrichment was greater under arable land cover than improved pasture: catchments with no agriculture had an average of 2 mg L^−1^ nitrate, increasing to 9·5 mg L^−1^ in catchments with 100% improved pasture and 14 m gL^−1^ in sites with 100% arable land cover. Therefore, we attribute the differences in invertebrate responses to arable and pasture land cover to the greater magnitude of nitrate enrichment from arable land cover. Nitrate adversely affects sensitive macroinvertebrates at concentrations exceeding 8·8 mg L^−1^, which we predicted in catchments with more than 50% arable land cover (Camargo, Alonso & Salamanca 2005). Unmeasured physicochemical changes, such as increased pesticide concentrations, may also have contributed to the decline in sensitive invertebrate taxa (Schriever *et al*. 2007).

We suggest that the magnitude of the nitrate enrichment from improved pasture, coupled with increases in light availability associated with riparian vegetation loss, had a subsidy effect on invertebrate communities through supplementation of autochthonous food resources (Liess *et al*. 2012). Although this analysis did not show the predicted increase in filamentous algae and macrophytes with nutrient enrichment, it is likely that these are insensitive indicators of in‐stream production and that elevated nutrients increased the nutritional quality of algae or the availability of epilithic algae for grazing invertebrates (Niyogi *et al*. 2007). Such subsidies often increase invertebrate abundance and, if pollution‐sensitive taxa have low abundances, rarefaction effects of this increased abundance could explain the observed increase in sensitive species representation with increased pastoral land cover, where nutrient levels were below the thresholds at which sensitivities are exceeded. Several studies have demonstrated a ‘subsidy–stress’ response with pastoral development, in which invertebrate richness increases with initial nutrient enrichment until a threshold beyond which further enrichment and excessive sedimentation result in reduced richness (Niyogi *et al*. 2007). The present results suggest that on average, current levels of pastoral intensity subsidize macroinvertebrate communities. The magnitude of this effect, an increase of six (catchment) and eight (riparian) families between sites with no improved pasture and 100% improved pasture land cover, is likely to have consequences for biotic interactions and ecosystem functioning. Further research is needed to determine the consequences of these changes in invertebrate communities and the intensity at which pastoral farming begins to deleteriously impact on macroinvertebrate diversity.

Although responses to agricultural land cover were largely similar in direction and magnitude whether land cover was measured at the riparian or catchment scale, nutrient concentrations showed slightly greater effect sizes at the catchment scale. This suggests the total contributing area is the best predictor of nutrient delivery (Roth, Allan & Erikson 1996), especially in areas where buffering from riparian vegetation is low, as predicted in agricultural sites. Conversely, macroinvertebrate responses to arable land cover were larger when land cover was measured at the riparian scale. This supports the results of both Richards *et al*. (1997) and Peterson *et al*. (2011) who found in‐stream biota to have stronger relationships with riparian land use than catchment‐scale land use, due to riparian scale measurements capturing effects with higher connectivity to the stream channel.

In summary, the approach here has furthered understanding gained from previous observational and manipulative studies by estimating the effect sizes of likely cause–effect relationships between changing proportions of agricultural land cover and key metrics of stream biological condition across a full range of natural complexity. This approach identifies the land management priority of reducing nutrient loading from arable farming and highlights the need for further research into the effects of pastoral land‐use intensity. More broadly, this analysis illustrates the potential of propensity modelling to resolve the effects of large‐scale ecosystem pressures with greater confidence, and thus to guide land‐use policy.

## Data accessibility

The data sets used in this manuscript are available in online supporting information.

## Supporting information


**Fig. S1.** Locations of River Habitat Survey and water chemistry/invertebrate monitoring sites.
**Fig. S2.** Distribution of sites, split into five groups based on modelled likelihood of having arable land cover.
**Fig. S3.** Distribution of sites, split into five groups based on modelled likelihood of having improved pasture land cover.

**Table S1.** Data used to create propensity scores.

**Table S2.** Data used in models of relationships between physical habitat and agricultural land cover.

**Table S3.** Data used in models of relationships between water quality and invertebrate response variables and agricultural land cover.

**Table S4.** Correlations between environmental covariates and treatment land covers across the whole data set and within propensity groups.
**Table S5.** Number of sites per propensity group.
**Table S6.** Estimated responses of river habitat characteristics to agricultural land cover with data set split into differing number of propensity groups.
**Table S7.** Estimated responses of water chemistry and invertebrate community variables to agricultural land cover with data set split into differing number of propensity groups.

## References

[jpe12586-bib-0001] Armitage, P.D. , Moss, D. , Wright, J.F. & Furse, M.T. (1983) The performance of a new Biological Water Quality Score System based on macroinvertebrates over a wide range of unpolluted running‐water sites. Water Research, 17, 333–347.

[jpe12586-bib-0002] Benjamin, D.J. (2003) Does 401(k) eligibility increase saving?: evidence from propensity score subclassification. Journal of Public Economics, 87, 1259–1290.

[jpe12586-bib-0003] Benjamini, Y. & Yekutieli, D. (2005) False discovery rate‐adjusted multiple confidence intervals for selected parameters. Journal of the American Statistical Association, 100, 71–81.

[jpe12586-bib-0004] Beyer, H. (2005) Hawth's analysis tools for ArcGIS (spatialecology.com/htools/overview.php) [15/07/2015].

[jpe12586-bib-0005] Bottrill, M.C. , Walsh, J.C. , Watson, J.E. , Joseph, L.N. , Ortega‐Argueta, A. & Possingham, H.P. (2011) Does recovery planning improve the status of threatened species?. Biological Conservation, 144, 1595–1601.

[jpe12586-bib-5000] British Geological Survey (2007) 1:50,000 [Shapefile geospatial data], Updated Sept 2007, British Geological Survey, UK. Using: EDINA Geology Digimap Service, (http://edina.ac.uk/digimap).

[jpe12586-bib-0006] Burdon, F.J. , McIntosh, A.R. & Harding, J.S. (2013) Habitat loss drives threshold response of benthic invertebrate communities to deposited sediment in agricultural streams. Ecological Applications, 5, 1036–1047. 10.1890/12-1190.1 23967573

[jpe12586-bib-0007] Camargo, J.A. , Alonso, A. & Salamanca, A. (2005) Nitrate toxicity to aquatic animals: a review with new data for freshwater invertebrates. Chemosphere, 58, 1255–1267. 15667845 10.1016/j.chemosphere.2004.10.044

[jpe12586-bib-0008] Carlisle, D.M. & Hawkins, C.P. (2008) Land use and the structure of western US stream invertebrate assemblages: predictive models and ecological traits. Journal of the North American Benthological Society, 27, 986–999.

[jpe12586-bib-0009] Chamberlain, D.E. , Fuller, R.J. , Bunce, R.G.H. , Duckworth, J.C. & Shrubb, M. (2000) Changes in the abundance of farmland birds in relation to the timing of agricultural intensification in England and Wales. Journal of Applied Ecology, 37, 771–788.

[jpe12586-bib-0010] Chessman, B.C. (2013) Do protected areas benefit freshwater species? A broad‐scale assessment for fish in Australia's Murray‐Darling Basin. Journal of Applied Ecology, 50, 969–976.

[jpe12586-bib-0011] Dale, V.H. & Polasky, S. (2007) Measures of the effects of agricultural practices on ecosystem services. Ecological Economics, 64, 286–296.

[jpe12586-bib-0012] Dehejia, R.H. & Wahba, S. (2002) Propensity score‐matching methods for non‐experimental causal studies. The Review of Economics and Statistics, 84, 151–161.

[jpe12586-bib-0013] Donald, P.F. , Sanderson, F.J. , Burfield, I.J. & van Bommel, F.P.J. (2006) Further evidence of continent‐wide impacts of agricultural intensification on European farmland birds, 1990–2000. Agriculture, Ecosystems and Environment, 116, 189–196.

[jpe12586-bib-0014] Dudgeon, D. , Arthington, A.H. , Gessner, M.O. , Kawabata, Z.‐I. , Knowler, D.J. , Leveque, C. *et al* (2006) Freshwater biodiversity: importance, threats, status and conservation challenges. Biological Reviews, 81, 163–182. 16336747 10.1017/S1464793105006950

[jpe12586-bib-0015] Emery, J.C. , Gurnell, A.M. , Clifford, N.J. , Petts, G.E. , Morrissey, I.P. & Soar, P.J. (2003) Classifying the hydraulic performance of riffle‐pool bedforms for habitat assessment and river rehabilitation design. River Research and Applications, 22, 533–549.

[jpe12586-bib-0016] Environment Agency (2003) River Habitat Survey Guidance Manual: 2003 version. Environment Agency, Bristol.

[jpe12586-bib-0017] Graham, M.H. (2003) Confronting multicollinearity in ecological multiple regression. Ecology, 84, 2809–2815.

[jpe12586-bib-0018] Guo, S. & Fraser, M.W. (2014) Propensity Score Analysis: Statistical Methods and Applications. Sage publications, London.

[jpe12586-bib-0019] Harrell, F.E. (2001) Regression Modeling Strategies. Springer‐Verlag, New York.

[jpe12586-bib-0020] Imai, K. & Van Dyk, D.A. (2004) Causal inference with general treatment regimes: generalizing the propensity score. Journal of the American Statistical Association, 99, 854–866.

[jpe12586-bib-0021] Knapp, A.K. , Smith, M.D. , Collins, S.L. , Zambatis, N. , Peel, M. , Emery, S. *et al* (2004) Generality in ecology: testing North American grassland rules in South African savannas. Frontiers in Ecology and the Environment, 2, 611–612.

[jpe12586-bib-0022] Kyriakeas, S.A. & Watzin, M.C. (2006) Effects of adjacent agricultural activities and watershed characteristics on stream macroinvertebrate communities. Journal of the American Water Resources Association, 42, 425–441.

[jpe12586-bib-0023] Larsen, S. & Ormerod, S.J. (2010) Combined effects of habitat modification on trait composition and species nestedness in river invertebrates. Biological Conservation, 143, 2638–2646.

[jpe12586-bib-0024] Lee, L. & Helsel, D. (2005) Statistical analysis of water‐quality data containing multiple detection limits: S‐language software for regression on order statistics. Computers and Geosciences, 31, 1241–1248.

[jpe12586-bib-0025] Liess, A. , LeGros, A. , Wagenhoff, A. , Townsend, C.R. & Matthaei, C.D. (2012) Landuse intensity in stream catchments affects the benthic food web: consequences for nutrient supply, periphyton C:nutrient ratios, and invertebrate richness and abundance. Freshwater Science, 31, 813–824.

[jpe12586-bib-0026] Matthaei, C. , Piggott, J. & Townsend, C. (2010) Multiple stressors in agricultural streams: interactions among sediment addition, nutrient enrichment and water abstraction. Journal of Applied Ecology, 47, 639–649.

[jpe12586-bib-0027] McDowell, R.W. , Drewry, J.J. , Muirhead, R.W. & Paton, R.J. (2003) Cattle treading and phosphorus and sediment loss in overland flow from grazed cropland. Australian Journal of Soil Research, 41, 1521–1532.

[jpe12586-bib-0028] Meador, M.R. & Goldstein, R.M. (2003) Assessing water quality at large geographic scales: relations among land use, water physicochemistry, riparian condition, and fish community structure. Environmental Management, 31, 504–517. 12677296 10.1007/s00267-002-2805-5

[jpe12586-bib-0029] Morton, D. , Rowland, C. , Wood, C. , Meek, L. , Marston, C. , Smith, G. & Simpson, I.C. (2011) Final report for LCM2007 – the new UK land cover map. CS Technical Report No 11/07 NERC/Centre for Ecology & Hydrology 108pp. (CEH project number: C03259).

[jpe12586-bib-0030] Murphy, J.F. & Davy‐Bowker, J. (2005) Spatial structure in lotic macroinvertebrate communities in England and Wales: relationship with physical, chemical and anthropogenic stress variables. Hydrobiologia, 534, 151–164.

[jpe12586-bib-0031] Murray‐Bligh, J. (1999) Procedures for Collecting and Analysing Macroinvertebrate Samples. Environment Agency, Bristol.

[jpe12586-bib-0032] Niyogi, D.K. , Koren, M. , Arbuckle, C.J. & Townsend, C.R. (2007) Stream communities along a catchment land use gradient: subsidy‐stress responses to pastoral development. Environmental Management, 39, 213–225. 17160511 10.1007/s00267-005-0310-3

[jpe12586-bib-0033] N imon, K. , Oswald, F. & Roberts, J.K. (2013). yhat: Interpreting Regression Effects. R package version 2.0‐0. http://CRAN.R-project.org/package=yhat [15/07/2015].

[jpe12586-bib-0034] Pebesma, E.J. (2004) Multivariable geostatistics in S: the gstat package. Computers and Geosciences, 30, 683–691.

[jpe12586-bib-0035] Perry, M. & Hollis, D. (2005) The generation of monthly gridded data sets for a range of climatic variables over the UK. International Journal of Climatology, 25, 1041–1054.

[jpe12586-bib-0036] Peterson, E.E. , Sheldon, F. , Darnell, R. , Bunn, S.E. & Harch, B.D. (2011) A comparison of spatially explicit landscape representation methods and their relationship to stream condition. Freshwater Biology, 56, 590–610.

[jpe12586-bib-0037] Quinn, J. & Hickey, C. (1990) Magnitude of effects of substrate particle size, recent flooding, and catchment development on benthic invertebrates in 88 New Zealand rivers. New Zealand Journal of Marine and Freshwater Research, 24, 411–427.

[jpe12586-bib-0039] Richards, C. , Haro, R.J. , Johnson, L.B. & Host, G.E. (1997) Catchment and reach‐scale properties as indicators of macroinvertebrate species traits. Freshwater Biology, 37, 219–230.

[jpe12586-bib-0040] Riley, R. , Townsend, C.R. , Niyogi, D.K. , Arbuckle, C.A. & Peacock, K.A. (2003) Headwater stream response to grassland agricultural development in New Zealand. New Zealand Journal of Marine and Freshwater Research, 37, 389–403.

[jpe12586-bib-0041] Robertson, G.P. & Swinton, S.M. (2005) Reconciling agricultural productivity and environmental integrity: a grand challenge for agriculture. Frontiers in Ecology and Environment, 3, 38–46.

[jpe12586-bib-0042] Robins, J.M. & Rotnitzky, A. (2001) Comment on “Inference for Semiparametric Models: some Questions and an Answer”, by J. P. Bickel and J. Kwon. Statistica Sinica, 11, 920–936.

[jpe12586-bib-0043] Robinson, R.A. & Sutherland, W.J. (2002) Changes in arable farming and biodiversity in Great Britain. Journal of Applied Ecology, 39, 157–176.

[jpe12586-bib-0044] Rosenbaum, P.R. (2002) Observational Studies, 2nd edn Springer, New York, New York, USA.

[jpe12586-bib-0045] Rosenbaum, P.R. & Rubin, D.B. (1983) The central role of the propensity score in observational studies for causal effects. Biometrika, 70, 41–55.

[jpe12586-bib-0046] Roth, N.E. , Allan, J.D. & Erikson, D.L. (1996) Landscape influences on stream biotic integrity assessed at multiple spatial scales. Landscape Ecology, 11, 141–156.

[jpe12586-bib-0047] Schmidt‐Kloiber, A. & Hering, D. (eds) (2012) The Ecology taxa and autecology database for freshwater organisms, version 5.0, www.freshwaterecology.info [14/11/2012].

[jpe12586-bib-0048] Schriever, C.A. , Ball, M.H. , Holmes, C.H. , Maud, S. & Liess, M. (2007) Agricultural intensity and landscape structure: influences on the macroinvertebrate assemblages of small streams in Northern Germany. Environmental Toxicology and Chemistry, 26, 346–357. 17713223 10.1897/05-629r.1

[jpe12586-bib-0049] Seager, K. , Baker, L. , Parsons, H. , Raven, P.J. & Vaughan, I.P. (2012) The rivers and streams of England and Wales: an overview of their physical character in 2007–2008 and changes since 1995–1996 River Conservation and Management (eds Boon P. & Raven P.J.), pp. 27–41. Wiley‐Blackwell, Chichester.

[jpe12586-bib-0050] Standing Committee of Analysts (1987) Kjeldahl Nitrogen in Waters. HMSO, London.

[jpe12586-bib-0051] Standing Committee of Analysts (1992) Phosphorus and Silicon in Waters. Effluents and Sludges, HMSO, London.

[jpe12586-bib-0052] Thompson, R.M. & Townsend, C.R. (2004) Land‐use influences on New Zealand stream communities – effects on species composition, functional organization and food‐web structure. New Zealand Journal of Marine and Freshwater Research, 38, 595–608.

[jpe12586-bib-0053] Tilman, D. , Balzer, C. , Hill, J. & Befort, B.L. (2011) Global food demand and the sustainable intensification of agriculture. Proceedings of the National Academy of Science USA, 108, 20260–20264. 10.1073/pnas.1116437108 PMC3250154 22106295

[jpe12586-bib-0054] Townsend, C. , Arbuckle, C. , Crowl, T. & Scarsbrook, M. (1997) The relationship between land use and physicochemistry, food resources and macroinvertebrate communities in tributaries of the Taieri River, New Zealand: a hierarchically scaled approach. Freshwater Biology, 37, 177–191.

[jpe12586-bib-0055] Van Sickle, J. (2003) Analyzing correlations between stream and watershed attributes. Journal of the American Water Resources Association, 39, 717–726.

[jpe12586-bib-0056] Vansteelandt, S. & Daniel, R.M. (2014) On regression adjustment for the propensity score. Statistics in Medicine, 33, 4053–4072. 24825821 10.1002/sim.6207

[jpe12586-bib-0057] Vaughan, I.P. , Merrix‐Jones, F.L. & Constantine, J.A. (2013) Successful predictions of river characteristics across England and Wales based on ordination. Geomorphology, 194, 121–131.

[jpe12586-bib-0058] Vaughan, I.P. & Ormerod, S.J. (2010) Linking ecological and hydromorphological data: approaches, challenges and future prospects for riverine science. Aquatic Conservation: Marine and Freshwater Ecosystems, 20, S125–S130.

[jpe12586-bib-0059] Vaughan, I.P. & Ormerod, S.J. (2012) Large‐scale, long‐term trends in British river macroinvertebrates. Global Change Biology, 18, 2184–2194.

[jpe12586-bib-0060] Vaughan, I.P. & Ormerod, S.J. (2014) Linking interdecadal changes in British river ecosystems to water quality and climatic dynamics. Global Change Biology, 20, 2725–2740. 24757015 10.1111/gcb.12616

[jpe12586-bib-0061] Weitzen, S. , Lapane, K.L. , Toledano, A.Y. , Hume, A.L. & Mor, V. (2005) Weaknesses of goodness‐of‐fit tests for evaluating propensity score models: the case of the omitted confounder. Pharmacoepidemiology and Drug Safety, 14, 227–238. 15386700 10.1002/pds.986

[jpe12586-bib-0062] Wood, S.N. (2006) On confidence intervals for generalized additive models based on penalized regression splines. Australian and New Zealand Journal of Statistics, 48, 445–464.

[jpe12586-bib-0063] Yuan, L. (2010) Estimating the effects of excess nutrients on stream invertebrates from observational data. Ecological Applications, 20, 110–125. 20349834 10.1890/08-1750.1

