# Supplementary material for: Resolving large‐scale pressures on species and ecosystems: propensity modelling identifies agricultural effects on streams
Source: J Appl Ecol. 2016 Jan 18;53(2):408–17. doi: 10.1111/1365-2664.12586 (PMC5102586; doi:10.1111/1365-2664.12586)
Supplement: Supplementary file 1 — Fig. S1. Locations of River Habitat Survey and water chemistry/invertebrate monitoring sites. Fig. S2. Distribution of sites, split into five groups based on modelled likelihood of having arable land cover. Fig. S3. Distribution of sites, split into five groups based on modelled likelihood of having improved pasture land cover. [file JPE-53-408-s001.docx]

**Supporting Information**


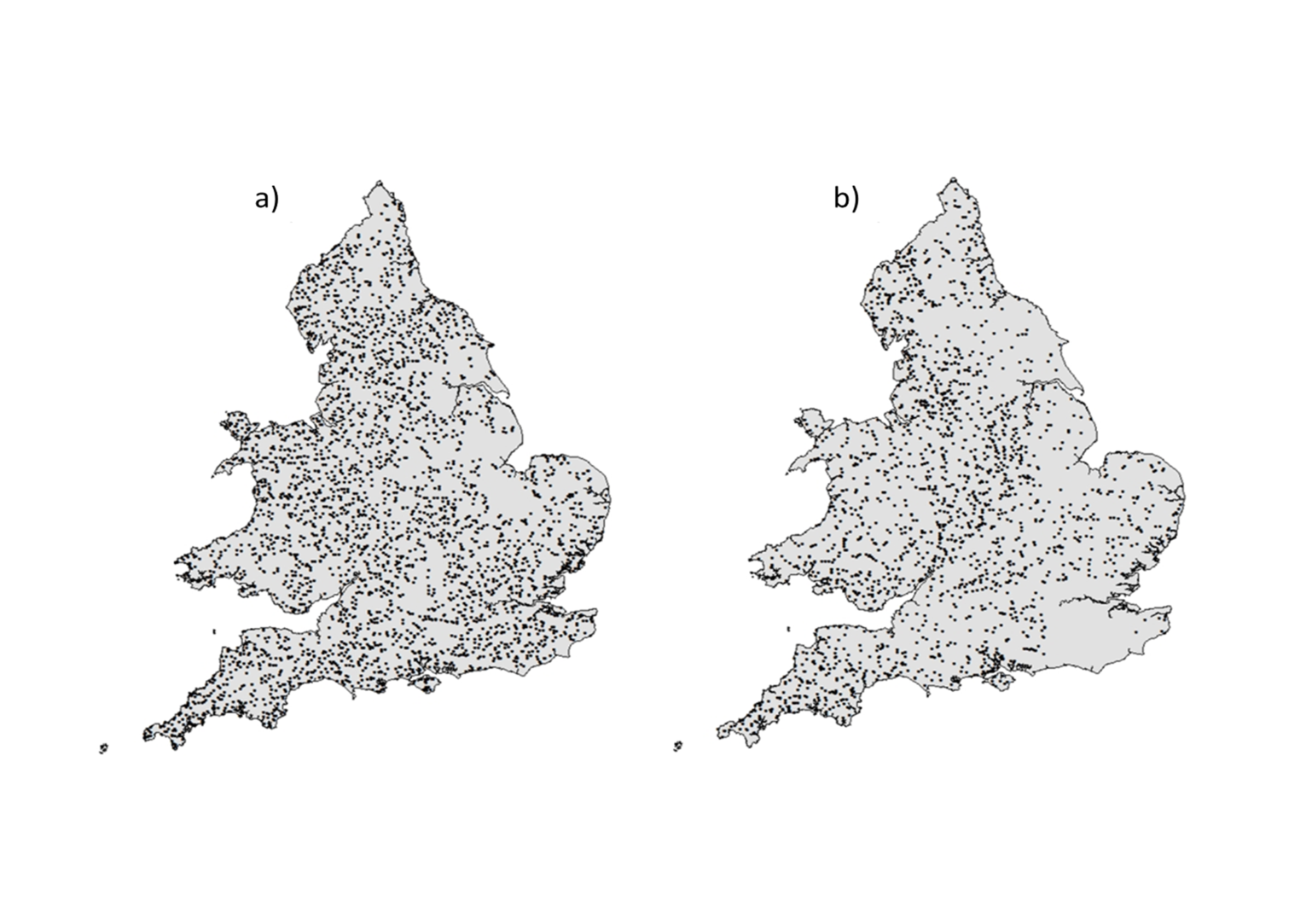


© Crown Copyright and Database Right 2015. Ordnance Survey (Digimap Licence).

*Figure S1 – Distribution of a) River Habitat Survey sites and b) Water chemistry/invertebrate monitoring sites used in analysis.*

*
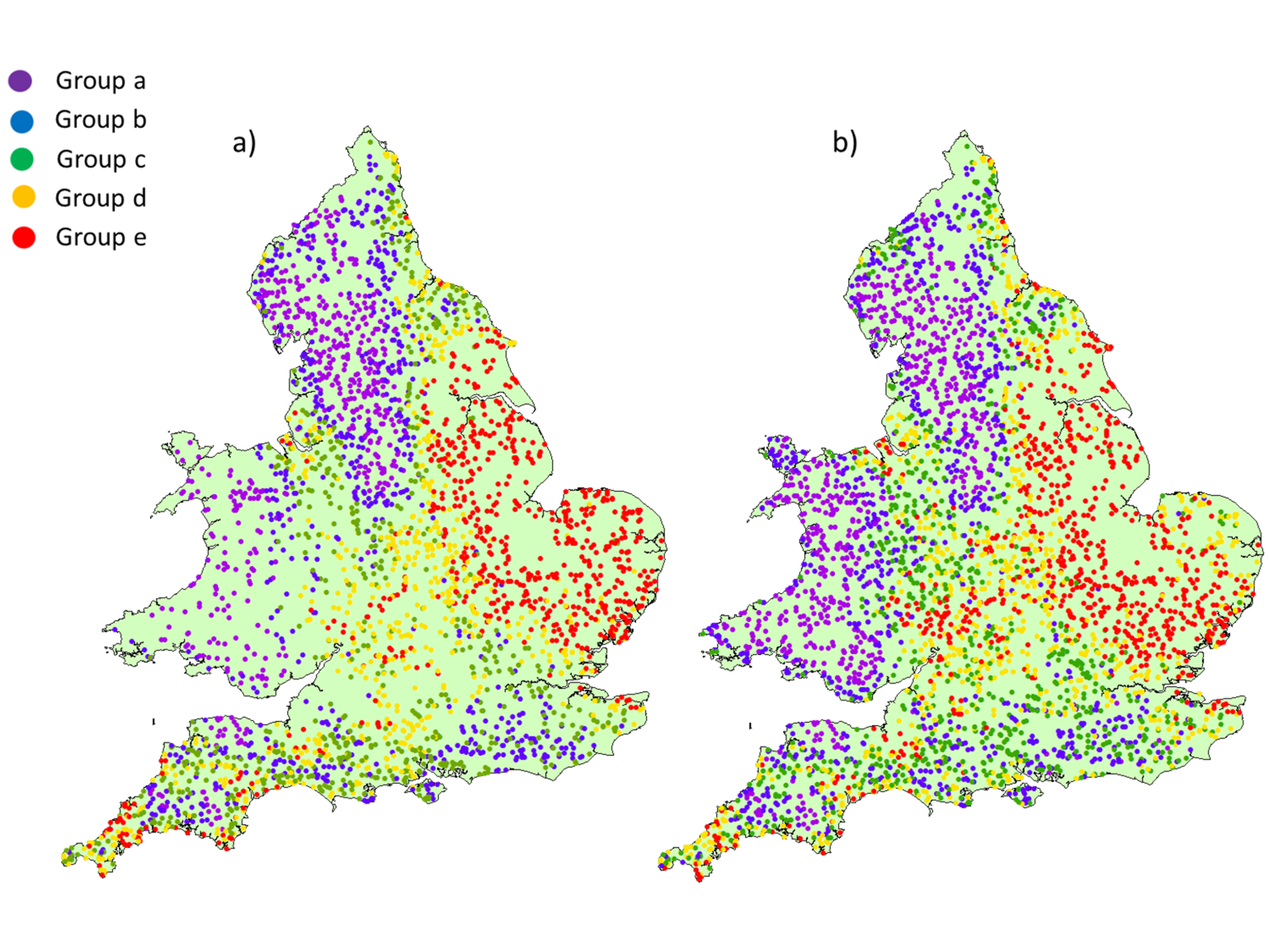
*

© Crown Copyright and Database Right 2015. Ordnance Survey (Digimap Licence).

*Figure S2 – Distribution of sites grouped by predicted proportion of arable land cover in a) the catchment and b) an 50 m upstream riparian strip. Predicted values are derived from the propensity model, based on climatic, locational and geological factors. Sites in group A have the lowest predicted proportion of arable land cover and sites in group E the highest.*


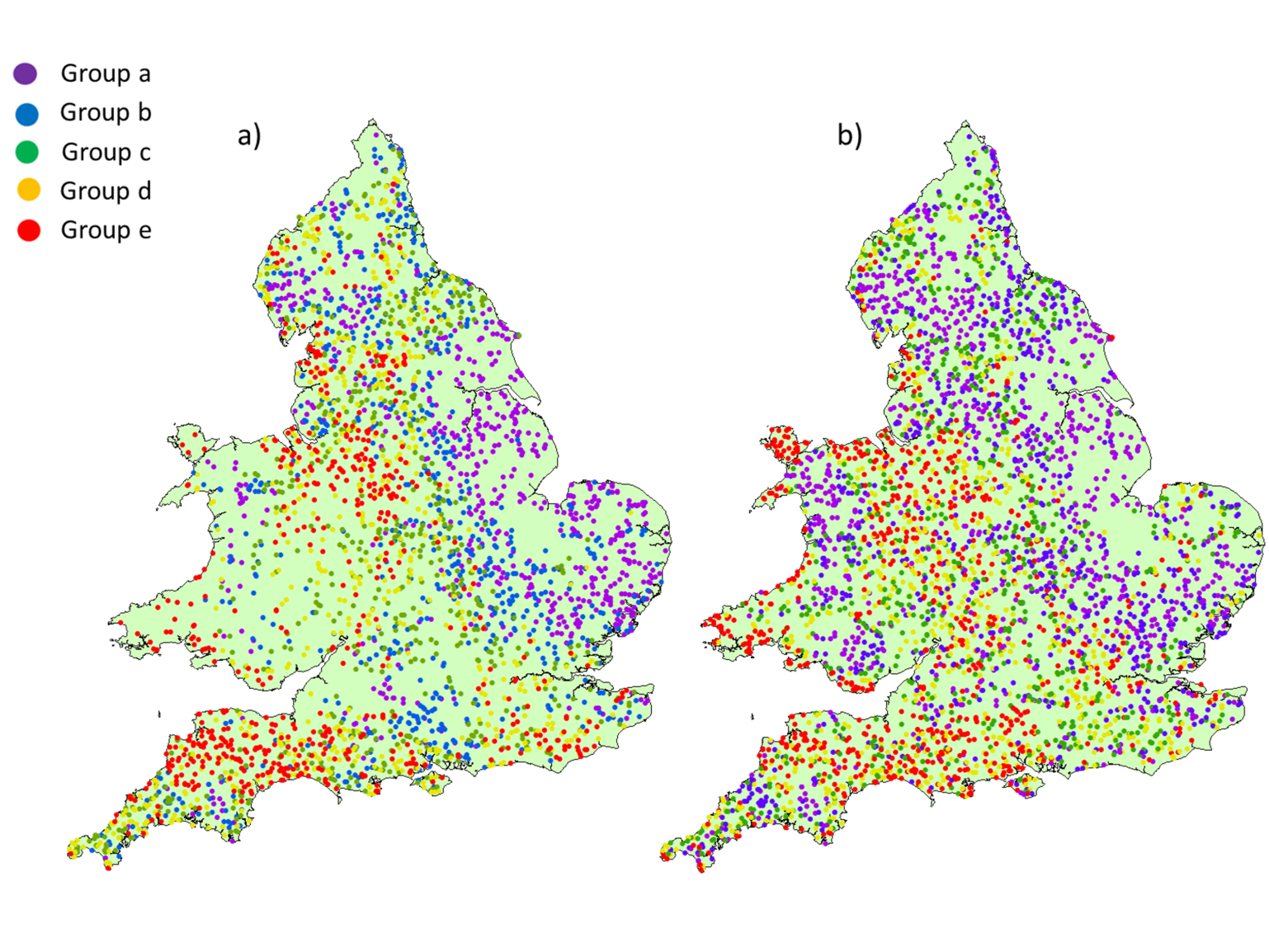


A)

© Crown Copyright and Database Right 2015. Ordnance Survey (Digimap Licence).

*Figure S3 – Distribution of sites grouped by predicted proportion of improved pasture land cover in a) the catchment and b) a 50 m upstream riparian strip. Predicted values are derived from the propensity model, based on climatic, locational and geological factors. Sites in group A have the lowest predicted proportion of improved pasture land cover and sites in group E the highest.*
