# Supplementary material for: Resolving large‐scale pressures on species and ecosystems: propensity modelling identifies agricultural effects on streams
Source: J Appl Ecol. 2016 Jan 18;53(2):408–17. doi: 10.1111/1365-2664.12586 (PMC5102586; doi:10.1111/1365-2664.12586)
Supplement: Supplementary file 5 — Table S4. Correlations between environmental covariates and treatment land covers across the whole data set and within propensity groups. Table S5. Number of sites per propensity group. Table S6. Estimated responses of river habitat characteristics to agricultural land cover with data set split into differing number of propensity groups. Table S7. Estimated responses of water chemistry and invertebrate community variables to agricultural land cover with data set split into differing number of propensity groups. [file JPE-53-408-s005.docx]

***Supplementary Tables***

*Table S4 – Number of sites per group after splitting the whole dataset into five groups based on percentiles of the propensity score for each land cover type then separating River Habitat Survey and water chemistry/invertebrate monitoring sites.*

| *Group* | River habitat survey sites | | | |  | Invertebrate/Chemistry sites | | | |
| --- | --- | --- | --- | --- | --- | --- | --- | --- | --- |
|  | *Improved catchment* | *Improved riparian strip* | *Arable catchment* | *Arable riparian strip* |  | *Improved catchment* | *Improved riparian strip* | *Arable catchment* | *Arable riparian strip* |
|  |  |  |  |  |  |  |  |  |  |
| A | 523 | 709 | 355 | 578 |  | 157 | 132 | 202 | 182 |
| B | 487 | 598 | 460 | 638 |  | 175 | 203 | 185 | 178 |
| C | 417 | 568 | 467 | 616 |  | 202 | 193 | 172 | 174 |
| D | 388 | 562 | 429 | 597 |  | 200 | 190 | 202 | 187 |
| E | 387 | 602 | 491 | 614 |  | 199 | 191 | 172 | 189 |
|  |  |  |  |  |  |  |  |  |  |

*Table S5 – Absolute magnitude of Pearson correlations coefficients between environmental covariates and treatment land covers across the whole data set and within propensity groups.*

|  | Arable catchment | |  | Arable  riparian strip | |  | Improved pasture catchment | |  | Improved pasture riparian strip | |
| --- | --- | --- | --- | --- | --- | --- | --- | --- | --- | --- | --- |
| Covariates | Whole data set | Group average |  | Whole data set | Group average |  | Whole data set | Group average |  | Whole data set | Group average |
| Altitude | 0.44 | 0.15 |  | 0.37 | 0.10 |  | 0.11 | 0.13 |  | 0.16 | 0.12 |
| Slope | 0.21 | 0.08 |  | 0.20 | 0.08 |  | 0.07 | 0.06 |  | 0.11 | 0.04 |
| Temperature | 0.46 | 0.24 |  | 0.39 | 0.23 |  | 0.15 | 0.19 |  | 0.22 | 0.13 |
| Rainfall | 0.62 | 0.13 |  | 0.56 | 0.12 |  | 0.03 | 0.13 |  | 0.15 | 0.08 |
| Easting | 0.46 | 0.08 |  | 0.41 | 0.10 |  | 0.29 | 0.08 |  | 0.13 | 0.05 |
| Northing | 0.20 | 0.19 |  | 0.10 | 0.16 |  | 0.14 | 0.15 |  | 0.16 | 0.01 |
| Other agricultural land cover | 0.30 | 0.23 |  | 0.24 | 0.17 |  | 0.30 | 0.09 |  | 0.24 | 0.10 |
| Urban land cover | 0.10 | 0.12 |  | 0.07 | 0.07 |  | 0.10 | 0.12 |  | 0.09 | 0.10 |
|  |  |  |  |  |  |  |  |  |  |  |  |

*Table S6- Modelled responses of river habitat characteristics to changes in the proportion of agricultural land cover in the catchment or upstream 50 m riparian strip with the data set split into 4, 5 or 6 strata based on percentiles of propensity scores and using the whole dataset (‘Direct models’). Displayed values are odds ratios, the change in likelihood of occurrence of the habitat feature for each percent increase in the proportion of the treatment land cover ± 95 % confidence limit values.*

| Land cover | Number of propensity groups | Macrophytes | Filamentous algae | Sand and silt deposits | Stable deposits | Bankside trees |
| --- | --- | --- | --- | --- | --- | --- |
| Improved pasture catchment | *5* | 0.996 ± 0.011 | 0.992 ± 0.008 | 1.018 ± 0.010 | 1.002 ± 0.008 | 1.002 ± 0.008 |
|  | *4* | 0.998 ± 0.011 | 0.993 ± 0.008 | 1.018 ± 0.009 | 1.003 ± 0.009 | 0.999 ± 0.009 |
|  | *6* | 0.999 ± 0.012 | 0.991 ± 0.009 | 1.018 ± 0.011 | 1.002 ± 0.009 | 1.002 ± 0.008 |
|  | *Direct* | 1.000 ± 0.008 | 0.992 ± 0.006 | 1.016 ± 0.006 | 1.009 ± 0.006 | 1.000 ± 0.005 |
| Improved pasture riparian strip | *5* | 1.005 ± 0.008 | 0.998 ± 0.006 | 1.014 ± 0.005 | 1.003 ± 0.005 | 0.993 ± 0.005 |
|  | *4* | 1.006 ± 0.007 | 0.998 ± 0.006 | 1.015 ± 0.006 | 1.003 ± 0.005 | 0.993 ± 0.005 |
|  | *6* | 1.006 ± 0.010 | 0.998 ± 0.006 | 1.015 ± 0.005 | 1.003 ± 0.006 | 0.993 ± 0.006 |
|  | *Direct* | 1.006 ± 0.006 | 0.997 ± 0.004 | 1.012 ± 0.004 | 1.002 ± 0.004 | 0.993 ± 0.004 |
| Arable catchment | *5* | 1.001 ± 0.020 | 0.999 ± 0.012 | 1.015 ± 0.010 | 0.989 ± 0.011 | 0.987 ± 0.010 |
|  | *4* | 1.004 ± 0.019 | 0.996 ± 0.011 | 1.015 ± 0.010 | 0.988 ± 0.009 | 0.980 ± 0.011 |
|  | *6* | 1.007 ± 0.042 | 0.996 ± 0.018 | 1.013 ± 0.015 | 0.986 ± 0.016 | 0.975 ± 0.019 |
|  | *Direct* | 1.014 ± 0.007 | 1.004± 0.005 | 1.013 ± 0.005 | 0.987 ± 0.005 | 0.984 ± 0.005 |
| Arable riparian strip | *5* | 0.990 ± 0.013 | 1.003 ± 0.009 | 1.019 ± 0.008 | 0.991 ± 0.008 | 0.984 ± 0.008 |
|  | *4* | 0.997 ± 0.012 | 1.001 ± 0.009 | 1.020 ± 0.008 | 0.991 ± 0.008 | 0.982 ± 0.008 |
|  | *6* | 0.995 ± 0.015 | 1.002 ± 0.010 | 1.019 ± 0.009 | 0.991 ± 0.009 | 0.983 ± 0.009 |
|  | *Direct* | 1.008 ± 0.005 | 1.001 ± 0.004 | 1.013 ± 0.004 | 0.988 ± 0.004 | 0.982 ± 0.004 |

*Table S7- Modelled responses of water chemistry and invertebrate community metrics to changes in the proportion of agricultural land cover in a site’s catchment or upstream 50 m riparian strip with the data set split into 4, 5 or 6 strata based on percentiles of propensity scores and using the whole dataset (‘Direct models’). Displayed values are change in response value for each percent increase in the proportion of the treatment land cover ± 95 % confidence limit values.*

| Land-cover | Number of propensity groups | log(Phosphate) | log(Total Oxidised Nitrogen) | Richness | ASPT | Feeding guild diversity |
| --- | --- | --- | --- | --- | --- | --- |
| Improved pasture catchment | *5* | 0.013 ± 0.012 | 0.014 ± 0.007 | 0.069 ± 0.047 | 0.001 ± 0.005 | 2.40e^-4^ ± 1.38e^-4^ |
|  | *4* | 0.013 ± 0.011 | 0.012 ± 0.007 | 0.083 ± 0.045 | 0.001 ± 0.005 | 2.08e^-4^ ± 1.48e^-4^ |
|  | *6* | 0.09 ± 0.012 | 0.013 ± 0.007 | 0.082 ± 0.049 | 0.002 ± 0.005 | 2.46e^-4^ ± 1.48e^-4^ |
|  | *Direct* | 0.010 ± 0.004 | 0.013 ± 0.002 | 0.075 ± 0.015 | 0.002 ± 0.002 | 1.17e^-4^ ± 4.28e^-5^ |
| Improved pasture riparian strip | *5* | 0.004 ± 0.009 | 0.007 ± 0.005 | 0.085 ± 0.036 | 0.005 ± 0.004 | 5.61e^-5^ ± 1.02e^-4^ |
|  | *4* | 0.007 ± 0.009 | 0.007 ± 0.005 | 0.072 ± 0.035 | 0.004 ± 0.004 | 5.75e^-5^ ± 1.07e^-4^ |
|  | *6* | 0.006 ± 0.009 | 0.007 ± 0.006 | 0.075 ± 0.037 | 0.004 ± 0.004 | 6.14e^-5^ ± 9.49e^-5^ |
|  | *Direct* | 0.006 ± 0.003 | 0.008 ± 0.002 | 0.084 ± 0.014 | 0.004 ± 0.002 | 6.72e^-5^ ± 3.89e^-5^ |
| Arable catchment | *5* | 0.012 ± 0.020 | 0.019 ± 0.014 | 0.00 ± 0.038 | -0.008 ± 0.007 | 6.81e^-5^ ± 1.92e^-4^ |
|  | *4* | 0.008 ± 0.010 | 0.017 ± 0.011 | 0.040 ± 0.058 | -0.008 ± 0.007 | 1.28e^-4^ ± 1.40e^-4^ |
|  | *6* | 0.020 ± 0.025 | 0.017 ± 0.018 | -0.025 ± 0.088 | -0.022 ± 0.012 | 8.85e^-5^ ± 2.44e^-4^ |
|  | *Direct* | 0.011 ± 0.004 | 0.016 ± 0.002 | 0.007 ± 0.015 | -0.007 ± 0.002 | 2.75e^-5^ ± 4.18e^-5^ |
| Arable riparian strip | *5* | 0.004 ± 0.016 | 0.012 ± 0.012 | 0.006 ± 0.059 | -0.011 ± 0.007 | -5.8e^-5^ ± 1.71e^-4^ |
|  | *4* | 0.002 ± 0.015 | 0.010 ± 0.011 | 0.016 ± 0.056 | -0.010 ± 0.007 | 5.74e^-5^ ± 1.39e^-4^ |
|  | *6* | 0.005 ± 0.017 | 0.012 ± 0.012 | 0.022 ± 0.058 | -0.010 ± 0.008 | 8.36e^-5^ ± 1.31e^-4^ |
|  | *Direct* | 0.013 ± 0.003 | 0.017 ± 0.002 | -0.021 ± 0.014 | -0.009 ± 0.002 | -7.3e^-5^ ± 3.82e^-4^ |
